# Trends and Core Competence Shifts in Nurses’ Infectious Disease Emergency Response Competence Across COVID-19 Pandemic Phases: Repeated Cross-Sectional Survey and Network Analysis

**DOI:** 10.2196/83281

**Published:** 2026-03-27

**Authors:** Jiagen Xiang, Qiuxia Liang, Yufei Lu, Meizhen Lin, Juan Liu, Hongman Li, Jingjing Cai, Yan Wu, Bingbing Li, Wenting Liu, Ming Yang, Xiaoning Sun, Yan Luo, Yibing Tan, Hairun Liu, Jiaying Li, Liming Lu, Zengjie Ye

**Affiliations:** 1 School of Nursing, Guangzhou University of Chinese Medicine, 232 Outer Ring East Road, Guangzhou University City, Panyu District, Guangzhou, Guangdong, 510006, China, 86 15914411786; 2 Medical College of Acu-Moxi and Rehabilitation, Guangzhou University of Chinese Medicine, Guangzhou, Guangdong, China; 3 Infection Control Office, Guangdong Provincial Hospital of Traditional Chinese Medicine, Guangzhou, Guangdong, China; 4 Department of General Medicine, Guangdong Provincial Hospital of Traditional Chinese Medicine, Guangzhou, Guangdong, China; 5 Cancer Hospital Chinese Academy of Medical Sciences, Beijing, China; 6 Center of Cognitive and Sleeping, The People’s Hospital of Guangxi Zhuang Autonomous Region, Nanning, China; 7 The Nethersole School of Nursing, Faculty of Medicine, Chinese University of Hong Kong, Hong Kong, China (Hong Kong); 8 School of Nursing, Guangzhou Medical University, Guangzhou, Guangdong, China

**Keywords:** infectious disease emergencies, response competences, emergency preparedness, health system resilience, propensity score matching, network analysis, COVID-19, nurses

## Abstract

**Background:**

The COVID-19 pandemic exposed structural vulnerabilities in the global health emergency workforce. Sustained monitoring of response competence dynamics is critical for maintaining health system resilience during protracted outbreaks.

**Objective:**

This study aimed to investigate trends and structural transitions in nurses’ infectious disease emergency response competence during different phases of the COVID-19 pandemic.

**Methods:**

Using the Healthcare Workers’ Infectious Disease Emergency Response Competence Questionnaire, 3 repeated cross-sectional surveys were conducted in February 2020, August 2021, and February 2023. The participants were matched in a 1:1:1 ratio by their demographic characteristics via propensity scores. Temporal trends were analyzed using ANOVA, and competence structure transitions were examined via network analysis.

**Results:**

Three-wave analyses (n=2525 per wave) demonstrated a sustained decline in competence, from 4.05 (SD 0.63) in February 2020 to 3.81 (SD 0.66) in August 2021 and further to 3.70 (SD 0.66) in February 2023. All pairwise comparisons were statistically significant (all *P*<.001). Network analysis identified critical structural shifts in competence architecture: in February 2020, the core network node was T24 (emergency management of body fluid exposure; strength=1.764), whereas in August 2021 and February 2023, the core network node was T19 (acquisition of key information on emerging infectious diseases; strength=1.759 and 1.852, respectively). Network structure comparisons revealed significant differences between February 2020 and August 2021 (*P*=.01) and between February 2020 and February 2023 (*P*=.01), whereas the difference between August 2021 and February 2023 was not significant (*P*=.07).

**Conclusions:**

Despite accumulated pandemic experience, nurses’ infectious disease response competence declined substantially, indicating systemic fragility during prolonged crises. However, this experience reshaped competence architecture, evolving from technical infection prevention toward higher-order competence in information integration and clinical decision-making under uncertainty. To rebuild resilience, phase-specific training programs are essential: early-phase training emphasizes infection prevention, whereas prolonged outbreaks focus on information identification and decision support. Additionally, standardized information platforms and psychological support are essential to manage ongoing pandemic pressures.

## Introduction

The threat of emerging infectious diseases has remained a persistent challenge to global public health, highlighting the critical need for robust health care system preparedness and response capabilities [1,2]. Nurses, who constitute the largest segment of the health care workforce, are indispensable at every stage of an outbreak. Their performance not only influences individual patient outcomes but also affects the resilience of the entire health care system [3,4]. Central to this role is the concept of infectious disease emergency response competence, defined as the integrated knowledge, skills, and experience required to deliver rapid and comprehensive responses during infectious disease crises [5,6].

The profound and prolonged experience of the COVID-19 pandemic compels us to learn how pandemics evolve through distinct phases. The World Health Organization has consequently advocated for phase-tailored strategies to underpin health system resilience [7]. Consequently, nursing emergency preparedness programs should be agile—continuously refined to match changes in epidemiology, resource availability, and clinical priorities [8]. Therefore, understanding how nurses’ infectious disease emergency response competence shifted across these pandemic phases is essential not only for interpreting the past but also, crucially, for preparing effective responses when the next inevitable threat emerges.

Existing studies have reported inconsistent levels of infectious disease emergency response competence among nurses as the COVID-19 pandemic has evolved [9-11]. These inconsistencies may stem from unexamined differences between pandemic phases. Although individual-level predictors such as educational attainment, prior training, and clinical experience have previously been identified [12-14], the contextual influence of distinct pandemic phases—each with its own operational challenges—has not been fully explored, particularly regarding sustained impacts over an extended duration such as during the COVID-19 pandemic.

Conceptually, infectious disease emergency response competence trajectories may reflect the interplay of two opposing forces: (1) potential competence enhancement through cumulative experiential learning as nurses gain practical exposure during prolonged outbreaks [5,14] and (2) potential competence erosion resulting from sustained psychological stress, physical exhaustion [15], and depleted cognitive resources [16]. Critically, beyond overall levels, the structure of required competence is also likely to undergo significant shifts during a prolonged crisis such as the COVID-19 pandemic.

However, empirical evidence mapping both the temporal trends and structural shifts in competence across distinct pandemic phases is lacking, and existing data do not capture these complex, longitudinal interactions. This critical gap impedes our understanding of how experiential learning and prolonged stress interact to shape both the overall level and the structural configuration of infectious disease emergency response competence over time. Therefore, this study aimed to examine temporal trends and structural transitions in nurses’ infectious disease emergency response competence across 3 COVID-19 pandemic phases. We hypothesized the following:

- Hypothesis 1: cumulative experiential learning will drive a progressive increase in overall infectious disease emergency response competence.
- Hypothesis 2: the configuration of core infectious disease emergency response competencies will shift to meet phase-specific demands.

To test these hypotheses, we conducted 3 repeated cross-sectional surveys of registered nurses in Guangdong Province across 3 pandemic phases (early surge, Delta containment, and post-Omicron transition). We used 1:1:1 propensity score matching to balance staffing characteristics and applied network analysis to decode structural transitions among core competencies. The results may help identify actionable intervention targets to support phase-tailored preparedness strategies.

## Methods

### Study Design

This study adopted a repeated cross-sectional survey design to investigate trends and structural transitions in nurses’ infectious disease emergency response competence across 3 key COVID-19 pandemic phases in Guangdong Province: February 2020 (early surge), August 2021 (Delta containment), and February 2023 (post-Omicron transition).

In February 2020, Guangdong reported a rapid surge in cases, overwhelmed health care capacity, and a strong resolve among medical staff to fight the pandemic despite limited resources [17]. By August 2021, Guangdong had implemented a “dynamic zero-COVID” policy that included mass testing and strict mobility controls, while the Delta outbreak tested these strategies and drove protocol standardization [18]. In February 2023, following the relaxation of restrictions, Guangdong faced another surge in cases at a time when many health care workers had already been infected or were themselves becoming infected, further intensifying the strain on the health care system. The focus shifted toward long-term mitigation and resilience strategies as the health care system adjusted to endemicity [19]. Together, these 3 waves capture contrasting, policy-relevant phases of the pandemic, providing a clear basis for examining phase-dependent competence dynamics. For a timeline of key epidemiological milestones, policy interventions, and their implications for nursing competence, please refer to Table S1 in Multimedia Appendix 1.

Participant recruitment used convenience sampling of registered nurses from Guangdong Province at each of the 3 time points. Consistent with the repeated cross-sectional design, no active tracking of individuals occurred across survey waves; each wave was treated as an independent sample despite the possibility of incidental reparticipation by some nurses.

### Participants

Eligible participants were registered nurses with a valid license, aged ≥18 years, with more than 1 year of frontline clinical experience, and who provided informed consent. We excluded nurses who were in training programs, completing internships, or on leave during the survey period.

The sample size was estimated using a precision-based approach for the total competence score: n = (Z₁–α/₂ × σ/δ)², where Z₁–α/₂=1.96 for a 95% confidence level. On the basis of a previous study [20] reporting a total score of 116.13 (SD 22.84), we set σ=22.84 and an absolute precision of *δ*=1 point on the total score scale. This yielded a required sample size of 2004. Assuming an 80% response rate, the target sample size was 2505 per wave. Although the sample size calculation was based on the total score, the final analysis and results in this study were reported using the mean score for clarity and ease of interpretation.

To address between-wave comparisons across 3 survey waves, we further applied Bonferroni adjustment for the 3 pairwise comparisons (*α*=.0167) and conducted an achieved power (sensitivity) analysis using the final propensity score–matched sample size (n=2525 per wave).

### Questionnaires

#### Demographic and Professional Characteristics

The participants’ demographic and professional characteristics, including age, gender, clinical experience, professional title, education level, and department, were selected on the basis of prior studies [20,21].

#### Healthcare Workers’ Infectious Disease Emergency Response Competence Questionnaire

The infectious disease emergency response competence questionnaire, developed by Kan et al [21] through a comprehensive literature review and expert consultation, is the most widely used assessment tool in China and has robust reliability and validity [10,12,22]. It comprises 36 items across 3 dimensions: emergency prevention competence (3 items), emergency preparedness competence (4 items), and emergency response competence (29 items). Each item is rated on a 5-point Likert scale (1=“totally unknown”; 5 =“very familiar”), yielding a total score ranging from 36 to 180. Higher scores indicate stronger competence. For descriptive interpretation, mean scores <3, 3 to 4, and >4 were considered low, moderate, and high perceived competence, respectively; these are interpretive anchors for the 5-point Likert scale rather than empirically validated clinical cutoffs. In this study, internal consistency was excellent, with Cronbach α coefficients ranging from 0.85 to 0.98 for the total scale and each dimension. The full 36-item list is provided in Table S2 in Multimedia Appendix 1.

### Data Collection

Surveys were administered via Wenjuanxing, a Chinese online survey platform. At each survey wave, the Wenjuanxing survey link and the recruitment invitation letter were first disseminated in a WeChat (Tencent Holdings Limited) working group of nursing department directors in Guangdong Province. Directors who agreed to participate forwarded the survey link, along with the standardized guidelines, to head nurses, who subsequently shared it with staff nurses. To ensure data integrity, each mobile number could submit the survey only once, and all items had to be completed before submission.

The link remained active for 7 days. If needed, brief reminders were sent via the same internal channels during the open period. After closure, the responses were screened for quality: those that failed the inclusion or exclusion criteria, were incomplete, or contained inconsistent answers, or were completed in under 120 seconds were excluded. Inconsistent answers referred to implausible or contradictory information in key demographic or professional fields (eg, clinical experience exceeding age-derived limits). The 120-second threshold was determined through pilot testing by the investigators as the conservative minimum time to complete the demographic items plus the 36 Likert scale items; responses below this threshold were considered insufficient engagement, raising concerns about data quality.

### Data Analysis

#### Propensity Score Matching

We used DecisionLinnc software (version 1.0; DecisionLinnc Core Team) [23,24] to perform propensity score matching (PSM) across 3 survey waves (February 2020, August 2021, and February 2023). Propensity scores were estimated using baseline covariates, including gender, age, professional title, education level, department, and clinical experience. A 2-stage matching approach was applied: first, matching wave 2020 vs wave 2021 using nearest-neighbor matching with a caliper of 0.25 SD of the logit-transformed propensity score, and then matching the pooled sample from stage 1 with wave 2023. A 1:1:1 matching ratio was achieved. Adequate balance was defined as a standardized mean difference (SMD) <0.10 for each covariate. We also conducted 3-wave comparisons and covariate-adjusted linear regression using the pre-PSM data to verify the robustness of the postmatching results.

#### Descriptive and Comparative Statistics

All analyses were conducted in R (version 4.4.3; R Foundation for Statistical Computing). Continuous variables with a normal distribution are presented as the mean (SD); nonnormally distributed continuous variables are reported as the median (IQR). Categorical variables are expressed as frequencies and percentages. Between-group comparisons for continuous variables were performed via Student 2-tailed *t* test or 1-way ANOVA, as appropriate; *χ*^2^ tests were used to assess differences in categorical variables. A 2-tailed *P*<.05 was considered statistically significant.

#### Psychometric Network Analysis

We estimated networks of the 36 items via the EBICglasso algorithm (bootnet package) and visualized them via the qgraph package. Each node’s predictability is indicated by a blue outer ring, and edge width represents association strength (green=positive correlation; red=negative correlation). Strength centrality was used to identify key nodes, as it is the most stable centrality measure [25]. In this context, strength centrality reflects the overall connectedness of an item to the rest of the network; items with higher strength can be interpreted as “hub” competencies that may exert broader influence on the competence architecture. Network stability was assessed via the correlation stability (CS) coefficient, with CS >0.50 indicating adequate stability [26]. We compared network structures across the 3 time points via the network comparison test to evaluate differences in both network structure and global strength.

### Ethical Considerations

This study was approved by the Ethics Board of the First Affiliated Hospital of Guangzhou University of Chinese Medicine (ZYYEC-ERK[2020]132). Informed consent was obtained electronically from all participants. Specifically, participants provided their consent by clicking the survey link, reviewing the electronic informed consent form (which detailed the study procedures, objectives, and their rights), and subsequently completing and submitting the questionnaire. This process strictly adhered to the principles of the Declaration of Helsinki. Participants were assured of data confidentiality and anonymity throughout the study. Participants did not receive any financial incentives or compensation for completing the survey.

## Results

### Participant Flow and Sample Size

**Figure 1.**
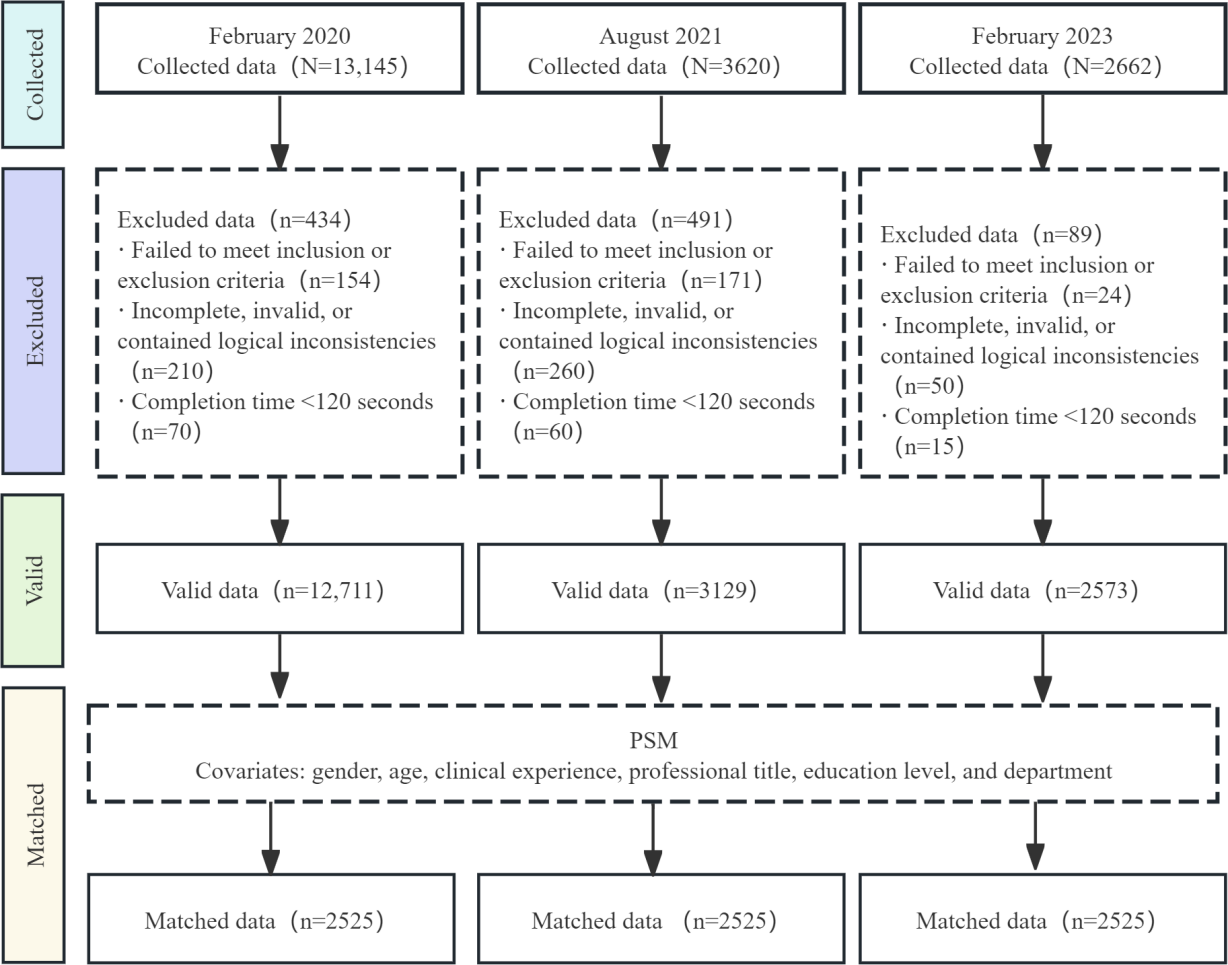
Flow diagram of participant enrollment, screening, and propensity score matching (PSM) for 3 repeated cross-sectional online surveys of registered nurses in Guangdong Province, China, conducted during 3 COVID-19 pandemic phases (February 2020, August 2021, and February 2023). The flowchart shows exclusions based on eligibility and data quality criteria and the 1:1:1 PSM procedure yielding 2525 nurses per wave.

Overall, the surveys yielded 12,711 valid responses in February 2020, 3129 in August 2021, and 2573 in February 2023. After PSM, each wave retained 2525 (19.86% for February 2020, 80.7% for August 2021, and 98.13% for February 2023) participants. The participant flow, screening, and matching procedures are detailed in Figure 1.

### Demographic and Professional Characteristics of Nurses After PSM

Table 1 presents the demographic and professional characteristics of the participants in each wave after PSM, which achieved adequate balance across covariates (all |SMD|<0.10; all *P*>.20). For example, the February 2023 group exhibited a mean age of 32.95 (SD 7.70) years; 96.63% (2440/2525) were female, 62.97% (1590/2525) held junior professional titles, and 62.22% (1571/2525) held a bachelor’s degree or higher, with 90.97% (2297/2525) working in nonemergency departments. Full prematching and postmatching characteristics and balance diagnostics (including SMDs) are provided in Table S3 in Multimedia Appendix 1.

**Table 1.** Demographic and professional characteristics of registered nurses in Guangdong Province, China, across 3 COVID-19 pandemic phases (February 2020, August 2021, and February 2023) after 1:1:1 propensity score matching (n=2525 per wave). Variables include gender, age, clinical experience, professional title, education level, and department. Between-wave balance is quantified using standardized mean differences (SMDs).

	February 2020	August 2021	February 2023	Chi-square (*df*)^a^	*P* value	SMD
Gender, n (%)				0.7 (2)	.70	0.015
Men	83 (3.29)	75 (2.97)	85 (3.37)			
Women	2442 (96.71)	2450 (97.03)	2440 (96.63)			
Age (years), mean (SD)^b^	32.93 (7.61)	32.64 (7.62)	32.95 (7.70)	—^c^	.27	0.027
Clinical experience (years), n (%)				0.5 (2)	.77	0.013
0‐5	622 (24.63)	643 (25.47)	626 (24.79)			
≥6	1903 (75.37)	1882 (74.53)	1899 (75.21)			
Professional title, n (%)				0.1 (4)	.99	0.005
Junior	1583 (62.69)	1591 (63.01)	1590 (62.97)			
Intermediate	768 (30.42)	761 (30.14)	763 (30.22)			
Senior	174 (6.89)	173 (6.85)	172 (6.81)			
Education level, n (%)				0.2 (2)	.92	0.008
Associate degree or below	946 (37.47)	940 (37.23)	954 (37.78)			
Bachelor’s degree or above	1579 (62.53)	1585 (62.77)	1571 (62.22)			
Department, n (%)				1.8 (2)	.42	0.024
Nonemergency	2302 (91.17)	2322 (91.96)	2297 (90.97)			
Emergency	223 (8.83)	203 (8.04)	228 (9.03)			

a Categorical variables were compared using chi-square tests. Chi-square values are reported to 1 decimal place, with df shown in parentheses.

b Age was compared using 1-way ANOVA (*F*_2,7572_=1.317).

c Not applicable.

### Nurses' Infectious Disease Emergency Response Competence

**Figure 2.**
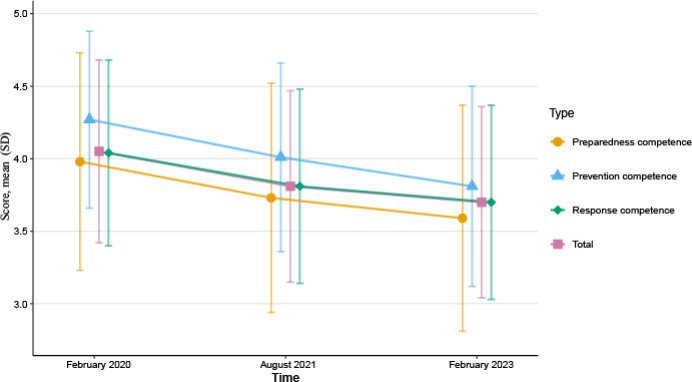
Mean (SD) total and domain scores of nurses’ infectious disease emergency response competence (36-item Healthcare Workers’ Infectious Disease Emergency Response Competence Questionnaire; 5-point Likert scale) among propensity score–matched registered nurses in Guangdong Province, China, across 3 COVID-19 pandemic phases (February 2020, August 2021, and February 2023; n=2525 per wave). Between-wave differences were tested using 1-way ANOVA with Bonferroni-adjusted pairwise comparisons.

The mean total score decreased from 4.05 (SD 0.63) in February 2020 to 3.81 (SD 0.66) in August 2021 and further to 3.70 (SD 0.66) in February 2023. The 3 competence domains (prevention, preparedness, and response) followed a similar downward trajectory. One-way ANOVA with Bonferroni correction demonstrated significant differences between every pair of time points for both total and domain scores (all *P*<.001). The detailed results are presented in Figure 2 and Tables S4 in Multimedia Appendix 1. Table S4A in Multimedia Appendix 1 summarizes the mean (SD) scores and overall comparisons across waves, and Table S4B in Multimedia Appendix 1 provides pairwise mean differences with 95% CIs, effect sizes (Cohen *d*), and percentage changes for each comparison. Sensitivity analyses using the unmatched sample (including 3-wave comparisons and covariate-adjusted regression) yielded consistent conclusions (Table S5 in Multimedia Appendix 1).

### Network Analysis of Nurses’ Infectious Disease Emergency Response Competence

#### Network Accuracy and Stability

**Figure 3.**
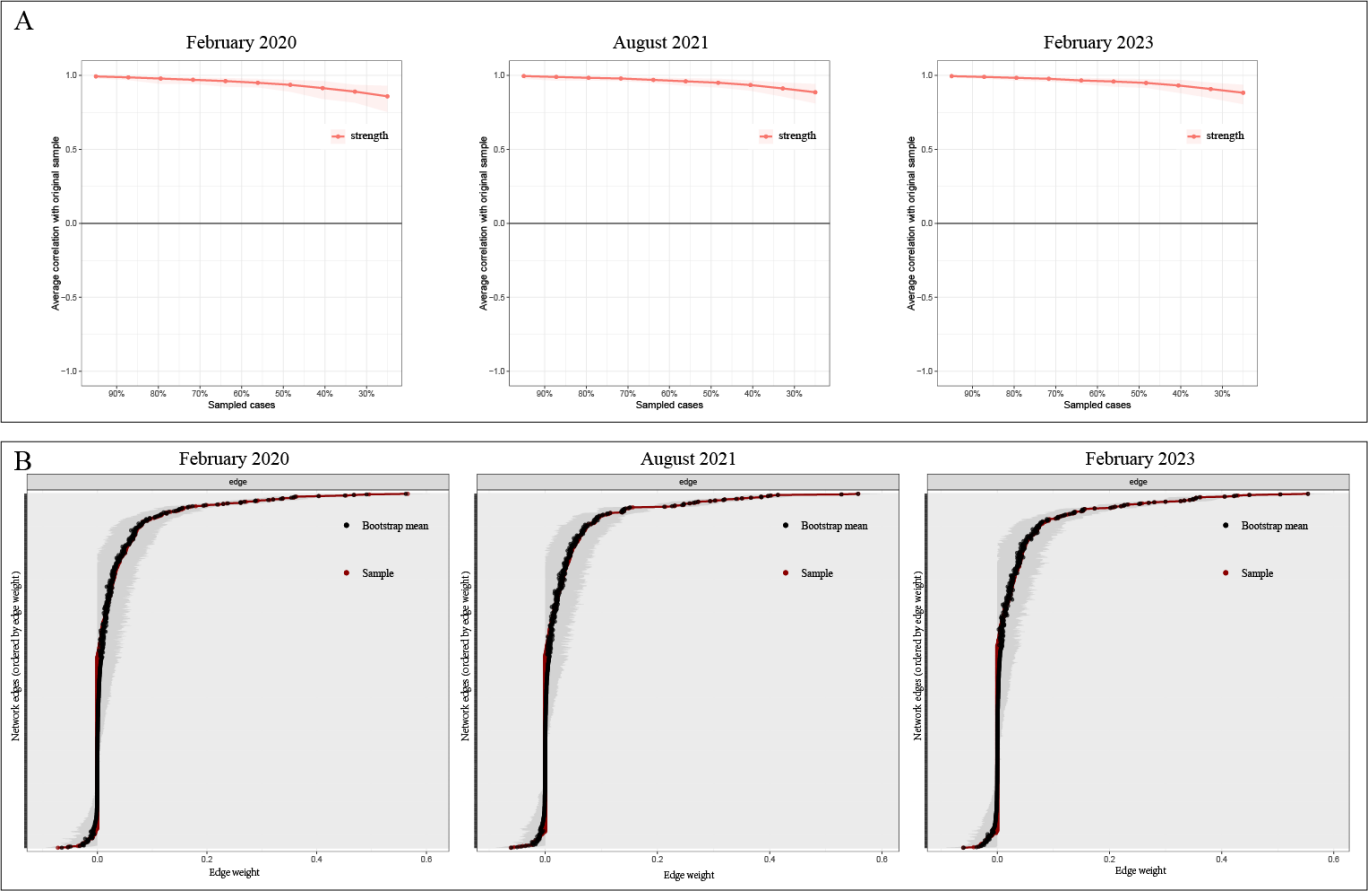
Accuracy and stability diagnostics for psychometric networks of nurses’ infectious disease emergency response competence in 3 COVID-19 pandemic phases among propensity score–matched registered nurses in Guangdong Province, China (February 2020, August 2021, and February 2023; n=2525 per wave). (A) Correlation stability (CS) coefficients for strength centrality (CS >0.50 indicates adequate stability for interpreting centrality). (B) Bootstrap 95% CIs (gray shading) for edge weights (narrower CIs indicate more precise edge-weight estimation).

We first evaluated the accuracy and stability of the estimated networks before interpreting network structure and centrality. These assessments supported the reliability of all 3 networks. First, the CS coefficient for strength centrality was 0.75 for each network, exceeding the recommended threshold of 0.5 (Figure 3A). Second, nonparametric bootstrapping indicated relatively narrow 95% CIs for edge weights, suggesting adequate precision in edge estimation (Figure 3B; gray bands indicate 95% CIs).

#### Network Structure and Centrality

**Figure 4.**
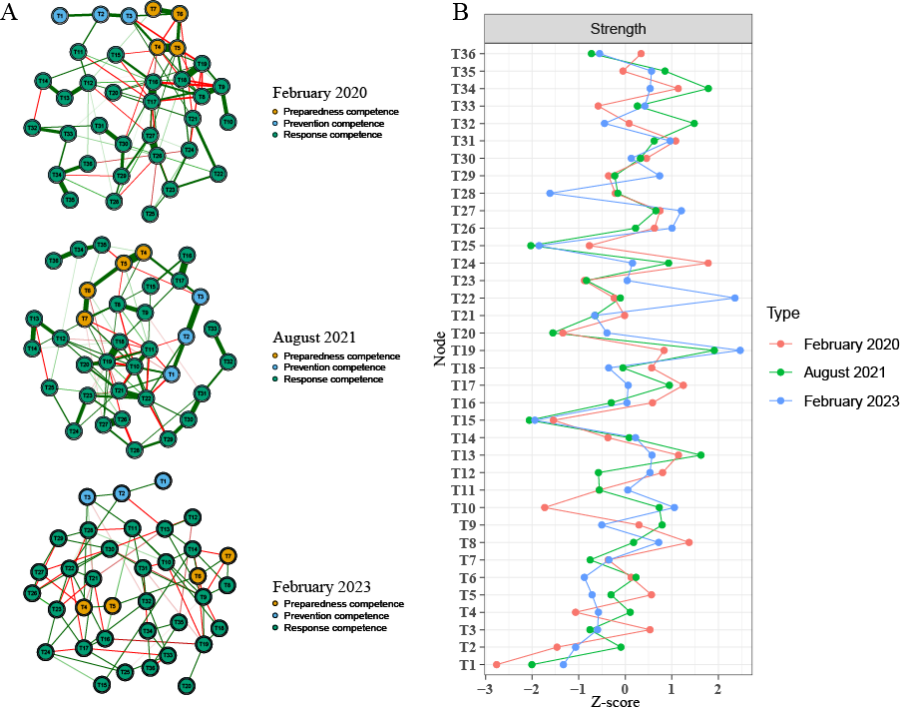
(A) Cross-sectional networks and (B) corresponding strength centralities of the infectious disease emergency response competence questionnaire among propensity score–matched registered nurses in Guangdong Province, China, across 3 COVID-19 pandemic phases (February 2020, August 2021, and February 2023; n=2525 per wave). Networks were estimated using EBICglasso; nodes represent items (T1-T36), and edges represent conditional associations (partial correlations), with thicker edges indicating stronger associations. Item-level strength centrality values for all nodes (T1-T36) across waves are provided in Table S2 in Multimedia Appendix 1.

On the basis of these stable networks, we examined node centrality and structural patterns. The range of strength centrality values across items was 1.125 to 1.764 in February 2020, 1.249 to 1.759 in August 2021, and 1.239 to 1.852 in February 2023. In February 2020, T24 (emergency management of body fluid exposure) showed the highest strength (1.764), whereas in August 2021 and February 2023, T19 (acquisition of key information on emerging infectious diseases) became the most central node (1.759 and 1.852, respectively). Cross-sectional networks and corresponding strength centralities are shown in Figure 4.

#### Network Comparison Tests

Network structure comparisons revealed significant differences between February 2020 and August 2021 (*P*=.01), and between February 2020 and February 2023 (*P*=.01), whereas the difference between August 2021 and February 2023 was not significant (*P*=.07). Global strength invariance tests indicated no significant differences in overall connectivity (*P*>.05). The results suggested that changes were mainly in the pattern or distribution of connections rather than the overall level of connectivity. Network-level summary indices and between-wave comparison metrics (including global strength, network correlations, and the proportion and number of significantly changed edges) are reported in Table S6 in Multimedia Appendix 1.

## Discussion

### Overview

This repeated cross-sectional study revealed 2 key findings: a statistically significant decline in nurses’ infectious disease emergency response competence over successive COVID-19 pandemic phases and a marked reconfiguration of core competence priorities. In contrast to our first hypothesis, overall infectious disease emergency response competence did not increase with pandemic experience; however, our second hypothesis was confirmed by a clear shift in structural centrality. Together, these results highlight systemic fragility in sustaining workforce capacity within the health emergency response system during prolonged crises and underscore the necessity of adaptive, phase-specific reinforcement strategies.

### Principal Findings

Contrary to expectations that experiential learning would bolster competence, scores for infectious disease emergency response competence declined steadily from February 2020 to February 2023. Nurses demonstrated above-average competence during the initial outbreak, which is consistent with prior findings [10,11,27]. This initial surge may be associated with short-term intensive training [10], strengthened professional motivation [28], and robust external support from governments and society [11,29]. However, these early gains were not sustained. The subsequent decline observed in later phases corresponded to patterns described in the Selye stress response theory [30], where prolonged exposure to pandemic stressors coincided with cumulative strain from excessive workloads, escalating burnout rates [31], and progressively diminishing training investments in later phases [8]. These sustained stressors may also contribute to cognitive depletion, reducing attentional and executive resources needed for continuous competence updating in rapidly changing clinical contexts [32]. Collectively, these factors may have counteracted potential competence gains from accumulated experience.

In addition, the observed decline may partly reflect changes in self-evaluation rather than true deterioration in clinical ability. As the pandemic progressed, clinical expectations expanded from basic infection control in the early phase to navigating multiple variants, evolving treatment pathways, vaccination strategies, and long-term management issues, potentially raising the benchmark for what nurses considered “competent” practice [33]. Under such shifting standards, increasing experience may also produce a form of learned humility (an inverse Dunning–Kruger pattern) [34], whereby greater awareness of uncertainty and knowledge gaps leads to more conservative self-assessments [35]. Consistent with this interpretation, our network results suggest a reorientation of competence priorities from technical execution (T24) toward higher-order cognitive competences in information acquisition, appraisal, and integration under uncertainty (T19). Taken together, the decline in self-reported competence likely reflects an interplay of cumulative stress and cognitive depletion, information burden, and recalibrated self-assessment standards rather than a single mechanism.

Beyond the quantitative decline that contradicted hypothesis 1, network analysis validated hypothesis 2 by revealing a structural priority shift—from T24 (emergency management of body fluid exposure) during the initial outbreak to T19 (acquisition of key information on emerging infectious diseases) in the Delta and Omicron phases. This transition signifies a fundamental evolution in competence architecture: from skill-based technical execution (T24) toward higher-order cognitive competence in information synthesis and clinical decision-making under uncertainty (T19).

In the initial phase, limited COVID-19 pandemic transmission knowledge, personal protective equipment shortages, and high mortality mandated rigorous self-protection [36]. The frontline workforce faced heightened infection risk from prolonged contact [37], compounded by fears of stigmatization and acute safety anxiety [38]. Consequently, T24—enhanced personal protective equipment training and exposure protocols—dominated, reflecting a survival-driven response to immediate biological threats. As the pandemic continued, competence demands transcended technical proficiency. Hyperdynamic informational uncertainty from variants and shifting policies created an infodemic [39,40]. Under the Simon bounded rationality model [41], rapidly shifting conditions and frequent guideline revisions strained decision-making. Rising caseloads and ambiguous protocols required real‐time information integration [3,42], elevating T19—the ability to rapidly acquire, filter, and contextualize key information—as the new core competence. This shift suggests that prolonged crises reprioritize competencies from procedural skills toward metacognitive capabilities for navigating complexity, and the observed decline in mean scores may partly reflect this reprioritization and a higher benchmark for competence in later phases rather than a uniform deterioration across all competency domains.

To address phase-specific challenges, we propose a tiered intervention framework: in the acute phase, prioritize biosafety through scenario-based training and secure personal protective equipment supply chains [10]. As the pandemic progresses, variant-driven policy turbulence requires the implementation of centralized information platforms (eg, Guangdong’s Yuekangma health code system) [43] and the establishment of rapid pandemic information integration systems, such as AI-assisted clinical decision support tools, to help nurses identify key guidance amidst information overload. During the sustained phase, integrate cyclic skill reinforcement programs that bridge the acute and recovery phases [8,44] and offer psychological support to mitigate chronic stress [16]. We provide a phase-specific implementation toolkit with actionable details in Table S7 in Multimedia Appendix 1. This phase-specific approach, aligned with pandemic dynamics, can mitigate competence erosion and increase health care system resilience during prolonged crises.

### Limitations

Several limitations warrant consideration. First, although we strengthened internal validity by applying 1:1:1 PSM to balance key staffing characteristics across waves, the repeated cross-sectional design precluded tracking individual nurses over time; residual confounding from time-varying contextual factors or unmeasured characteristics may remain. Second, participants were recruited only from Guangdong Province, where local policies and health system resources may differ from those in other regions, which may limit generalizability. Third, competence was assessed via self-reported measures, which may be influenced by reporting bias or changing self-evaluation standards as clinical expectations evolved. Fourth, most participants were from nonemergency departments; although this reflects workforce composition, the findings may not fully represent nurses in core outbreak-response units (eg, emergency department, intensive care unit, or infection control). Finally, focusing on nurses alone excludes other frontline professions (eg, physicians and public health specialists), constraining inferences about interdisciplinary preparedness.

### Recommendations for Further Research

While it is universally desirable that pandemics like the COVID-19 pandemic never recur, preparedness necessitates proactive scholarship. Future research should therefore use longitudinal cohort designs to track the same cohort of nurses across pandemic phases, thereby decoding individual competence trajectories through repeated measures. Integrating mixed methods approaches could reveal how stress, experiential learning, and systemic supports interact to drive competence changes, ultimately informing personalized prevention strategies. Where feasible, future studies should triangulate self-reported competence with objective or performance-based indicators and evaluate the implementation and effectiveness of phase-specific training, information support, and psychological interventions across diverse regions and professional groups.

### Conclusions

This study reveals a progressive decline in nurses’ infectious disease emergency response competence across 3 years of COVID-19 pandemic response, alongside a shift in core competence from technical infection control tasks to information acquisition and integration. These findings suggest that experience alone may not sustain preparedness during prolonged crises and underscore the need for phase-specific training, robust information infrastructure, and sustained psychological support to mitigate competence erosion and strengthen workforce resilience.

## Supplementary material

10.2196/83281 Multimedia Appendix 1 Supplementary tables detailing the COVID-19 pandemic timeline and nursing competence implications, the full 36-item competence questionnaire, participant characteristics before and after propensity score matching, wave-by-wave competence score comparisons, sensitivity analyses, network comparison metrics, and a phase-specific implementation toolkit.

## Abbreviations

PSM: propensity score matching
SMD: standardized mean difference
CS: correlation stability
