# Supplementary material for: Trends and Core Competence Shifts in Nurses’ Infectious Disease Emergency Response Competence Across COVID-19 Pandemic Phases: Repeated Cross-Sectional Survey and Network Analysis
Source: JMIR Public Health Surveill. 2026 Mar 27;12:e83281. doi: 10.2196/83281 (PMC13026426; doi:10.2196/83281)
Supplement: Multimedia Appendix 1 [file publichealth-v12-e83281-s001.pdf]

Supplementary Table S1. Timeline of key epidemiological milestones and policy interventions related to COVID-19 in Guangdong Province, China, and their implications for nursing infectious disease emergency response competence across three survey waves (February 2020, August 2021, and February 2023).

Date	Epidemiological/Variant Milestone	Key Policy/Healthcare Operations	Implications for Nursing Competence
January 2020	First confirmed case in Guangdong	Initial containment measures; no standardized protocols	Healthcare workers faced high uncertainty with minimal preparation for an unknown virus
February 2020	Rapid surge in cases (>1,000 cases)	Emergency response escalation, lockdown measures, PPE shortages	Healthcare workers faced overwhelming patient numbers, limited resources, and high uncertainty; despite challenges, strong resolve and dedication emerged among medical staff
May 2021	Delta variant detected in Guangdong	"Dynamic zero-COVID" policy implemented: mass testing, targeted lockdowns	Nurses had to quickly adapt to stricter protocols, and greater flexibility was required in responding to the rapidly changing pandemic
August 2021	Major Delta outbreak in Guangzhou	Mass testing, quarantine, contact tracing, strict mobility control	Under intense pressure, nurses operated by adhering to standardized emergency protocols, managing a large-scale crisis with limited training time
December 2022	Relaxation of non-pharmaceutical interventions (NPIs), nationwide policy shift	Transition from strict containment to mitigation strategies (Omicron surge)	Healthcare workers faced frequent policy changes; many had been infected and recovered, needing to cope with new variants of the virus
February 2023	Widespread Omicron infections in Guangdong	Policy relaxation, shift to endemic management and risk stratification	After three years of sustained pressure, healthcare workers adapted to frequent policy changes while meeting new patient demands, demonstrating great resilience

Supplementary Table S2. Item list and item-level strength centrality values for the 36-item infectious disease emergency response competence questionnaire across three repeated cross-sectional survey waves of registered nurses in Guangdong Province, China (February 2020, August 2021, and February 2023; propensity score–matched sample, n=2525 per wave).

Items	Strength centrality value		
	Feb.2020	Aug.2021	Feb.2023
**Prevention competence**			
T1. Infection spectrum of infectious diseases (concepts and implications of incubation	1.125	1.256	1.324
T2. Transmission mechanisms of infectious diseases	1.307	1.502	1.361
T3. Principles of prevention and control of infectious diseases	1.588	1.416	1.426
**Preparedness competence**			
T4. Roles and responsibilities of healthcare workers in National Emergency Response Plan	1.364	1.527	1.429
T5. Responsibilities of medical staff under National Emergency Response Plan for Public	1.593	1.474	1.410
T6. Regulations on Public Health Emergencies	1.531	1.543	1.387
T7. Law of the People's Republic of China on Prevention and Control of Infectious	1.462	1.417	1.460
**Response competence**			
T8. Significance of symptom monitoring	1.707	1.537	1.609
T9. Definition of syndrome and target disease	1.555	1.615	1.439
T10.Classification of patients according to established case definitions in emergencies	1.270	1.608	1.655
T11.Time limit for reporting legal infectious diseases	1.435	1.442	1.516
T12. Correct completion of Infectious Disease Report Card	1.627	1.440	1.583
T13. Scope of information in infectious disease emergency reporting	1.674	1.723	1.589
T14. Reporting process for public health emergencies	1.461	1.525	1.539
T15. Basic knowledge of medical response to infectious disease emergencies	1.297	1.249	1.239
T16. Correct specimen collection for infectious disease patients	1.596	1.475	1.514
T17. Precautions for specimen preservation and transportation	1.689	1.635	1.517
T18. Identification of credible information sources during emerging infectious disease	1.594	1.507	1.459
T19. Acquisition of key information on emerging infectious diseases	1.631	1.759	1.852
T20. Psychological self-adjustment methods during emergencies	1.325	1.314	1.454
T21. Core principles of standard precautions	1.512	1.430	1.418
T22. Protection requirements for different transmission routes	1.480	1.500	1.836
T23. Correct donning and doffing of personal protective equipment	1.390	1.407	1.514
T24. Emergency management of body fluid exposure	1.764	1.633	1.530
T25. Principles for establishing infectious disease wards	1.405	1.253	1.251
T26. Proper hand hygiene implementation	1.602	1.542	1.648
T27. Proper disposal of medical waste for infectious diseases	1.619	1.598	1.676
T28. Properly disposing death bodies of potential and actual infectious disease patients	1.484	1.493	1.284
T29. Environmental disinfection methods	1.463	1.484	1.611
T30. Isolation principles for different transmission routes	1.578	1.556	1.527
T31. Implementation of patient isolation for infectious diseases	1.666	1.594	1.643
T32. Quarantine methods for close contacts of infectious diseases	1.525	1.704	1.447
T33. Development of health education programs for infectious disease control	1.432	1.547	1.567
T34. Response to bioterrorism attacks	1.674	1.743	1.583
T35. Emergency response to post-disaster infectious disease outbreaks	1.507	1.623	1.587
T36. Considerations for participation in international rescue operations	1.562	1.420	1.432

[Note] Each item is scored on a five-point Likert scale (1 represents "totally unknown" and 5 represents "very familiar").

Supplementary Table S3. Baseline characteristics of registered nurses in Guangdong Province, China, before and after 1:1:1 propensity score matching across three repeated cross-sectional COVID-19 survey waves (February 2020, August 2021, and February 2023). Balance diagnostics are presented using standardized mean differences (SMDs).

Variable	Level	Before PSM					After PSM				
		Feb 2020 (N=12,711)	Aug 2021 (N=3,129)	Feb 2023 (N=2,573)	*P*	SMD	Feb 2020 (N=2,525)	Aug 2021 (N=2,525)	Feb 2023 (N=2,525)	*P*	SMD
Gender	Male, n (%)	556 (4.37)	82 (2.62)	93 (3.61)	<.001	0.064	83 (3.29)	75 (2.97)	85 (3.37)	.7	0.015
	Female, n (%)	12,155 (95.63)	3,047 (97.38)	2,480 (96.39)			2,442 (96.71)	2,450 (97.03)	2,440 (96.63)		
Clinical experience (years)	0-5, n (%)	4,823 (37.94)	911 (29.11)	631 (24.52)	<.001	0.195	622 (24.63)	643 (25.47)	626 (24.79)	.769	0.013
	≥6, n (%)	7,888 (62.06)	2,218 (70.89)	1,942 (75.48)			1,903 (75.37)	1,882 (74.53)	1,899 (75.21)		
Age (years), mean(SD)		31.05(7.78)	32.22(7.90)	33.02(7.78)	<.001	0.169	32.93(7.61)	32.64(7.62)	32.95(7.70)	.268	0.027
Professional title	Junior, n (%)	9,261 (72.86)	2,040 (65.20)	1,603 (62.30)	<.001	0.161	1,583 (62.69)	1,591 (63.01)	1,590 (62.97)	.999	0.005
	Intermediate, n (%)	2,924 (23.00)	863 (27.58)	785 (30.51)			768 (30.42)	761 (30.14)	763 (30.22)		
	Senior, n (%)	526 (4.14)	226 (7.22)	185 (7.19)			174 (6.89)	173 (6.85)	172 (6.81)		
Education level	Associate degree or below, n (%)	6,745 (53.06)	1,432 (45.77)	973 (37.82)	<.001	0.206	946 (37.47)	940 (37.23)	954 (37.78)	.920	0.008
	Bachelor degree or above, n (%)	5,966 (46.94)	1,697 (54.23)	1,600 (62.18)			1,579 (62.53)	1,585 (62.77)	1,571 (62.22)		
Department	Non-emergency department, n (%)	11,769 (92.59)	2,907 (92.91)	2,323 (90.28)	<.001	0.063	2,302 (91.17)	2,322 (91.96)	2,297 (90.97)	.415	0.024
	Emergency department, n (%)	942 (7.41)	222 (7.09)	250 (9.72)			223 (8.83)	203 (8.04)	228 (9.03)		

Notes: P values are from one-way ANOVA (continuous variables) or chi-square tests (categorical variables). SMD values are reported as provided by the analysis output; SMD <0.10 is commonly considered indicative of acceptable balance.

Supplementary Table S4A. Domain and total scores of nurses' infectious disease emergency response competence across three repeated cross-sectional COVID-19 survey waves in Guangdong Province, China (February 2020, August 2021, and February 2023; propensity score–matched sample, n=2525 per wave). Values are presented as mean (SD).

Dimensional and total scores	Feb 2020 (N=2525)	Aug 2021 (N=2525)	Feb 2023 (N=2525)	*F*	*P*	Effect size η²[95% CI]	Cohen's f (95% CI]
Prevention competence, mean(SD)	4.27(0.61)	4.01(0.65)	3.81(0.69)	309.472	<.001*	0.076[0.065–0.087]	0.286[0.263-0.309]
Preparedness competence, mean(SD)	3.98(0.75)	3.73(0.79)	3.59(0.78)	164.79	<.001*	0.042[0.033–0.051]	0.209[0.186-0.231]
Response competence, mean(SD)	4.04(0.64)	3.81(0.67)	3.7(0.67)	168.964	<.001*	0.043[0.034–0.052]	0.211[0.188-0.234]
Total	4.05(0.63)	3.81(0.66)	3.7(0.66)	190.229	<.001*	0.048[0.039–0.057]	0.224[0.201-0.247]

Note: Data were normally distributed. * denotes that all comparisons showed statistically significant differences (one-way ANOVA with Bonferroni correction, *P* <.001). Effect sizes are reported as η² and Cohen's f with 95% confidence intervals.

Supplementary Table S4B. Pairwise comparisons of nurses' infectious disease emergency response competence scores across three repeated cross-sectional COVID-19 survey waves in Guangdong Province, China (February 2020, August 2021, and February 2023; propensity score–matched sample, n=2525 per wave). Results include mean differences with 95% confidence intervals, effect sizes (Cohen's d), and percentage changes.

Outcome	Comparison	Mean difference	95% CI	Effect size (Cohen's d)	Achieved power	% change from earlier
**Prevention competence**	Feb 2020 → Aug 2021	-0.26	[-0.29, -0.23]	-0.41	>99.99%	-6.1%
Prevention competence	Aug 2021 → Feb 2023	-0.20	[-0.24, -0.16]	-0.30	>99.99%	-5.0%
Prevention competence	Feb 2020 → Feb 2023	-0.46	[-0.50, -0.42]	-0.71	>99.99%	-10.8%
**Preparedness competence**	Feb 2020 → Aug 2021	-0.25	[-0.29, -0.21]	-0.32	>99.99%	-6.3%
Preparedness competence	Aug 2021 → Feb 2023	-0.14	[-0.18, -0.10]	-0.18	>99.99%	-3.8%
Preparedness competence	Feb 2020 → Feb 2023	-0.39	[-0.43, -0.35]	-0.51	>99.99%	-9.8%
**Response competence**	Feb 2020 → Aug 2021	-0.23	[-0.27, -0.19]	-0.35	>99.99%	-5.7%
Response competence	Aug 2021 → Feb 2023	-0.11	[-0.15, -0.07]	-0.16	99.97%	-2.9%
Response competence	Feb 2020 → Feb 2023	-0.34	[-0.38, -0.30]	-0.52	>99.99%	-8.4%
**Total**	Feb 2020 → Aug 2021	-0.24	[-0.28, -0.20]	-0.37	>99.99%	-5.9%
Total	Aug 2021 → Feb 2023	-0.11	[-0.15, -0.07]	-0.17	99.98%	-2.9%
Total	Feb 2020 → Feb 2023	-0.35	[-0.39, -0.31]	-0.54	>99.99%	-8.6%

Notes: 95% CIs were computed from group means and SDs[normal approximation) for descriptive interpretation. Cohen's d was calculated using pooled SD. Achieved power for each pairwise comparison was computed using a two-sample t test with Bonferroni-adjusted $\alpha = 0.0167$[three comparisons; n = 2525 per wave). Abbreviation: PSM, propensity score matching.

Supplementary Table S5A. Domain and total competence scores across three repeated cross-sectional COVID-19 survey waves in Guangdong Province, China (February 2020, August 2021, and February 2023; pre–propensity score matching sample).

**Competence scores, mean (SD)**	**Feb 2020 N=12711**	**Aug 2021 N=3129**	**Feb 2023 N=2573**	**F**	***P***	**η^2**
Prevention competence, mean(SD)	4.11(0.64)	4.02(0.65)	3.81(0.69)	232.051	<.001	0.025
Preparedness competence, mean(SD)	3.76(0.8)	3.73(0.8)	3.59(0.78)	46.862	<.001	0.005
Response competence, mean(SD)	3.83(0.67)	3.81(0.68)	3.70(0.67)	36.968	<.001	0.004
Total	3.84(0.66)	3.82(0.67)	3.70(0.66)	50.79	<.001	0.005

Notes: Scores are presented as mean item scores (range 1–5). Overall differences across the three waves were assessed using one-way ANOVA. η^2 indicates the proportion of variance explained by the survey wave (effect size).

Supplementary Table S5B. Pairwise comparisons of competence scores across three repeated cross-sectional COVID-19 survey waves in Guangdong Province, China (February 2020, August 2021, and February 2023; pre–propensity score matching sample).

Outcome	Comparison	Mean difference	95% CI	Effect size (Cohen's d)	% change from earlier	Significant (Bonferroni α=0.0167)
Prevention competence	Feb 2020→Aug 2021	-0.09	-0.12 to -0.07	-0.14	-2.3%	Yes
Prevention competence	Aug 2021→Feb 2023	-0.21	-0.24 to -0.17	-0.31	-5.2%	Yes
Prevention competence	Feb 2020→Feb 2023	-0.30	-0.33 to -0.27	-0.46	-7.3%	Yes
Preparedness competence	Feb 2020→Aug 2021	-0.03	-0.06 to 0.00	-0.04	-0.8%	No
Preparedness competence	Aug 2021→Feb 2023	-0.14	-0.18 to -0.1	-0.17	-3.7%	Yes
Preparedness competence	Feb 2020→Feb 2023	-0.17	-0.2 to -0.13	-0.21	-4.4%	Yes
Response competence	Feb 2020→Aug 2021	-0.01	-0.04 to 0.01	-0.02	-0.3%	No
Response competence	Aug 2021→Feb 2023	-0.11	-0.15 to -0.08	-0.17	-2.9%	Yes
Response competence	Feb 2020→Feb 2023	-0.12	-0.15 to -0.1	-0.19	-3.3%	Yes
Total	Feb 2020→Aug 2021	-0.02	-0.05 to 0.00	-0.03	-0.6%	No
Total	Aug 2021→Feb 2023	-0.12	-0.16 to -0.09	-0.18	-3.2%	Yes
Total	Feb 2020→Feb 2023	-0.14	-0.17 to -0.12	-0.22	-3.8%	Yes

Notes: 95% CIs were computed using an independent-samples t distribution with pooled SD. Cohen's d was calculated using pooled SD. Significance was assessed using a Bonferroni-adjusted α=0.0167 for three pairwise comparisons within each outcome

Supplementary Table S5C. Multivariable linear regression (ANCOVA) examining between-wave differences in nurses' infectious disease emergency response competence across three repeated cross-sectional COVID-19 survey waves in Guangdong Province, China (February 2020, August 2021, and February 2023; pre–propensity score matching sample), adjusting for gender, age, clinical experience, education level, professional title, and department.

Outcome (Y)	N	Aug 2021 vs Feb 2020 β (95% CI)	*P*	Feb 2023 vs Feb 2020 β (95% CI)	*P*	Feb 2023 vs Aug 2021 β (95% CI)	*P*	Overall P for Wave
Overall competence	18413	-0.019 (-0.045 to 0.007)	.147	-0.143 (-0.171 to -0.115)	<.001	-0.124 (-0.159 to -0.09)	<.001	<.001
Preparedness competence	18413	-0.029 (-0.06 to 0.002)	.066	-0.171 (-0.205 to -0.137)	<.001	-0.141 (-0.183 to -0.1)	<.001	<.001
Prevention competence	18413	-0.106 (-0.131 to -0.08)	<.001	-0.322 (-0.35 to -0.294)	<.001	-0.216 (-0.25 to -0.182)	<.001	<.001
Response competence	18413	-0.009 (-0.035 to 0.018)	.510	-0.121 (-0.15 to -0.092)	<.001	-0.112 (-0.147 to -0.077)	<.001	<.001

Notes: Each outcome was analyzed in a separate linear regression model with Wave entered as a categorical predictor (reference = Feb 2020) and the 6 baseline covariates included as adjustment variables. β represents the adjusted mean difference (later minus earlier); negative values indicate lower competence in the later wave. Overall P for Wave was obtained from the omnibus test of the Wave term in the adjusted model.

Supplementary Table S6. Network-level indices and between-wave comparison metrics for psychometric networks of nurses' infectious disease emergency response competence across three repeated cross-sectional COVID-19 survey waves in Guangdong Province, China (February 2020, August 2021, and February 2023; propensity score–matched sample, n=2525 per wave).

Section	Item	Feb 2020	Aug 2021	Feb 2023	Feb 2020 vs Aug 2021	Feb 2020 vs Feb 2023	Aug 2021 vs Feb 2023
**Network indices**	Clustering coefficient	0.375	0.276	0.349	—	—	—
	Density	0.076	0.07	0.075	—	—	—
	Global strength	27.247	27.246	27.158	—	—	—
	Average path length	1.954	1.944	2.021	—	—	—
	Mean predictability (R^2), mean (range)	0.718, 0.734(0.542-0.829)	0.727, 0.754(0.606-0.814)	0.757, 0.768(0.652-0.823)			
**Between-wave comparisons**	Network correlation (r)	—	—	—	0.839	0.834	0.88
	Significant change edge ratio (%)	—	—	—	11.6% (73/630)	12.9% (81/630)	4.4% (28/630)

Note.：Threshold = 0.05. Global strength is the sum of edge weights (Σ edge weights). Significant change edge ratio is shown as percentage with counts (changed edges / total tested edges).

Supplementary Table S7. Phase-specific implementation toolkit for sustaining nurses' infectious disease emergency response competence across three COVID-19 phases in Guangdong Province, China (February 2020, August 2021, and February 2023), including recommended training focuses, information-support strategies, and psychological support actions.

Pandemic phase	Training focus and format (minimum viable package)	Information infrastructure (minimum viable package)	Psychological support (minimum viable package)	Monitoring and evaluation (examples)
Early phase (outbreak emergence)	• Core IPC and PPE donning/doffing; exposure management (e.g., body fluid exposure)<br>• Just-in-time microlearning (5–10 min modules) + brief hands-on drills/simulation for high-risk procedures<br>• Daily/shift safety huddles to reinforce key updates and workflow changes	• Single authoritative portal for protocols (mobile-friendly)<br>• Clear version control and "what's new" change log<br>• Targeted push alerts for critical updates (role/unit specific)	• Rapid access to psychological first aid and confidential support channel<br>• Peer support point-of-contact within unit (brief check-ins)<br>• Normalize help-seeking through leadership messaging	• Completion/attendance tracking for required modules<br>• Direct observation checklists for PPE/exposure procedures<br>• Brief pre/post quizzes on critical updates
Middle phase (sustained transmission; policy/variant shifts)	• Information literacy and evidence appraisal; decision-making under uncertainty<br>• Case-based learning (regular brief sessions) + targeted refreshers when protocols change<br>• Peer learning/community-of-practice to share practical adaptations	• Searchable protocol repository with tagged content (unit/role)<br>• Weekly digest summarizing changes + links to full guidance<br>• Governance: designated clinical editor group to review and harmonize updates	• Periodic screening for distress/burnout risk (low-burden, confidential)<br>• Tiered support pathway: self-help resources → peer support → professional services<br>• Work-rest protection where feasible (micro-breaks, schedule flexibility)	• Pulse surveys on information usability and confidence<br>• Audit of protocol adherence for selected high-risk workflows<br>• Trend monitoring of competence scores by wave/unit (where available)
Late phase (endemic transition; prolonged response)	• Adaptive expertise, systems thinking, leadership/mentorship, career sustainability<br>• Self-directed learning plans with periodic review + optional workshops<br>• Mentorship/knowledge-transfer activities to sustain capability over time	• Archived protocols and rationale for changes (learning from prior versions)<br>• Optional decision-support tools embedded in routine systems (e.g., EHR links)<br>• Low-noise alerting to prevent fatigue (prioritize high-importance changes)	• Sustained mental health services with easy access and confidentiality<br>• Structured peer support and mentorship to reduce isolation<br>• Organizational interventions targeting chronic workload and retention	• Longitudinal tracking of competence (periodic assessments)<br>• Staff retention/turnover and sick leave indicators (contextual signals)<br>• Qualitative feedback on barriers to maintaining competence (focus groups/interviews)

Abbreviations: IPC, infection prevention and control; PPE, personal protective equipment; EHR, electronic health record.
